# Supplementary figures and images for: Structure, dynamics and free energy studies on the effect of point mutations on SARS-CoV-2 spike protein binding with ACE2 receptor
Source: PLoS One. 2023 Oct 5;18(10):e0289432. doi: 10.1371/journal.pone.0289432 (PMC10553274; doi:10.1371/journal.pone.0289432)

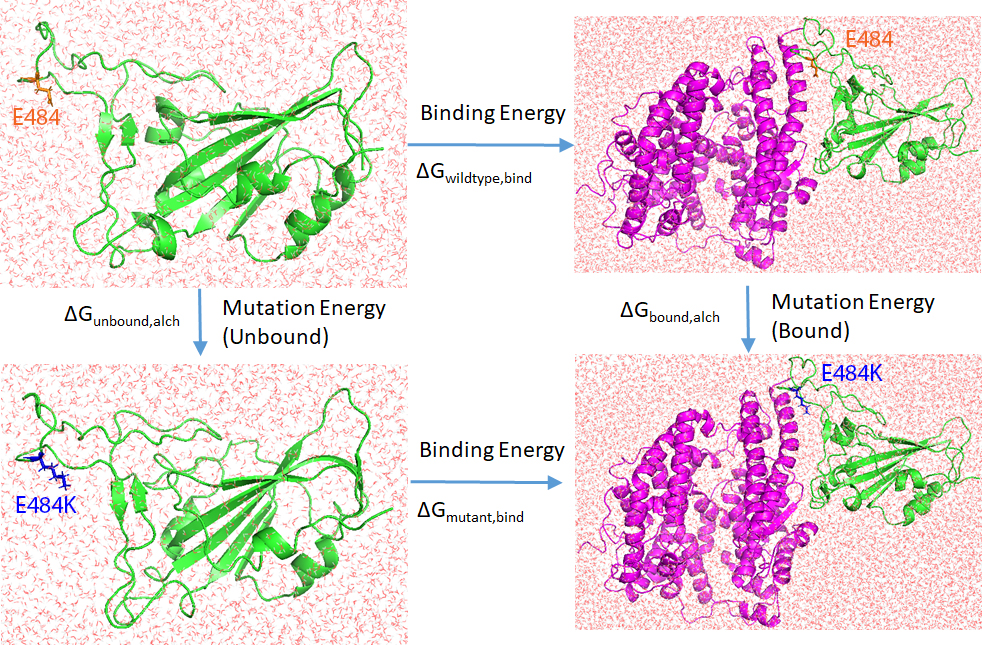

Supplement: S1 Fig — After mutating E484 into K484, it is shown in blue sticks. The RBD is shown in green cartoon while ACE2 shown in magenta cartoon. Water molecules are shown in red sticks and ions are not shown. (TIF) [file pone.0289432.s001.tif]

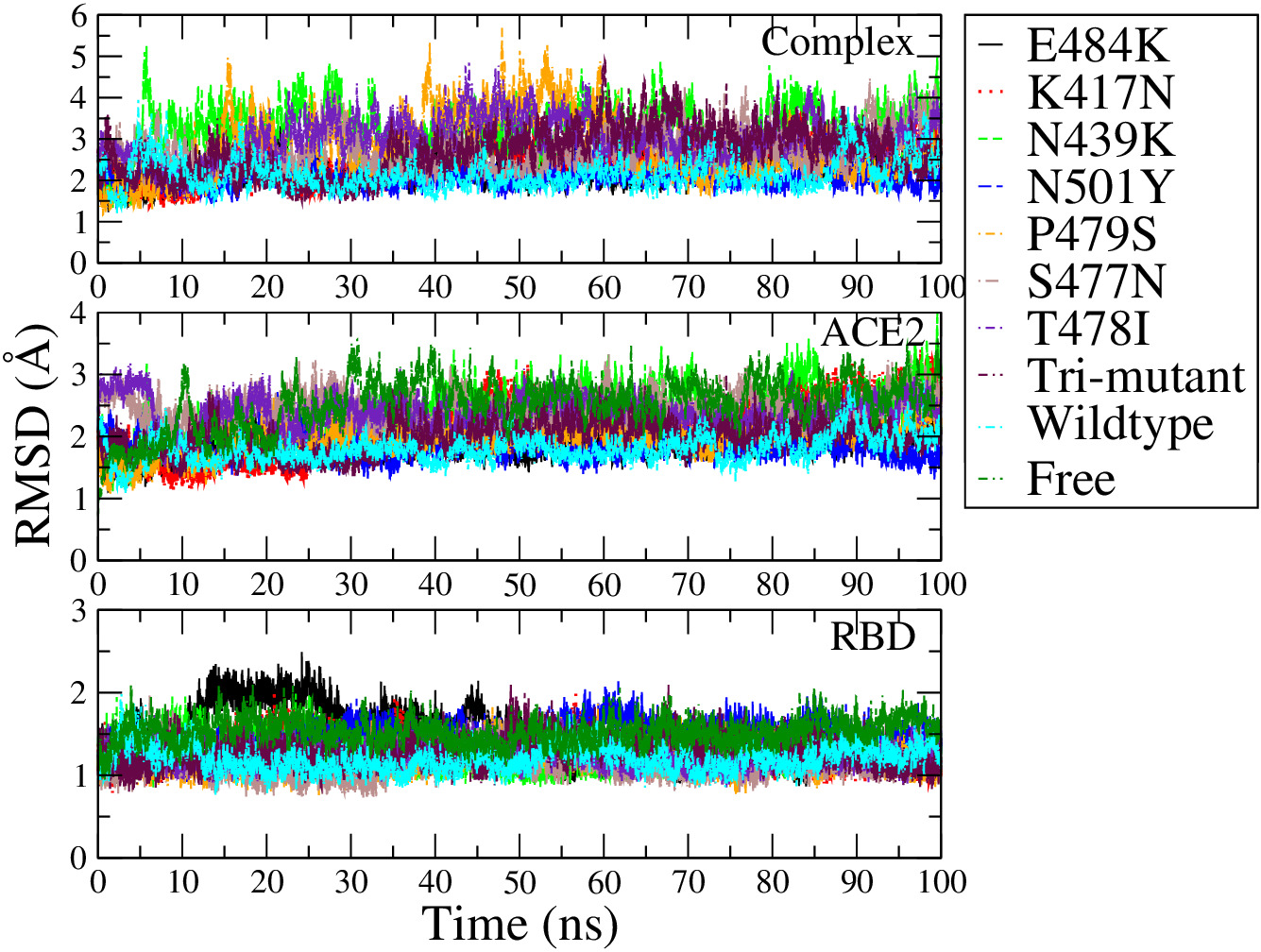

Supplement: S2 Fig — RMSD of ACE2&RBD mutant complexes (Top row), ACE2 (Middle row) and RBD (Bottom row) during 100 ns all-atom NAMD simulations. (TIF) [file pone.0289432.s002.tif]

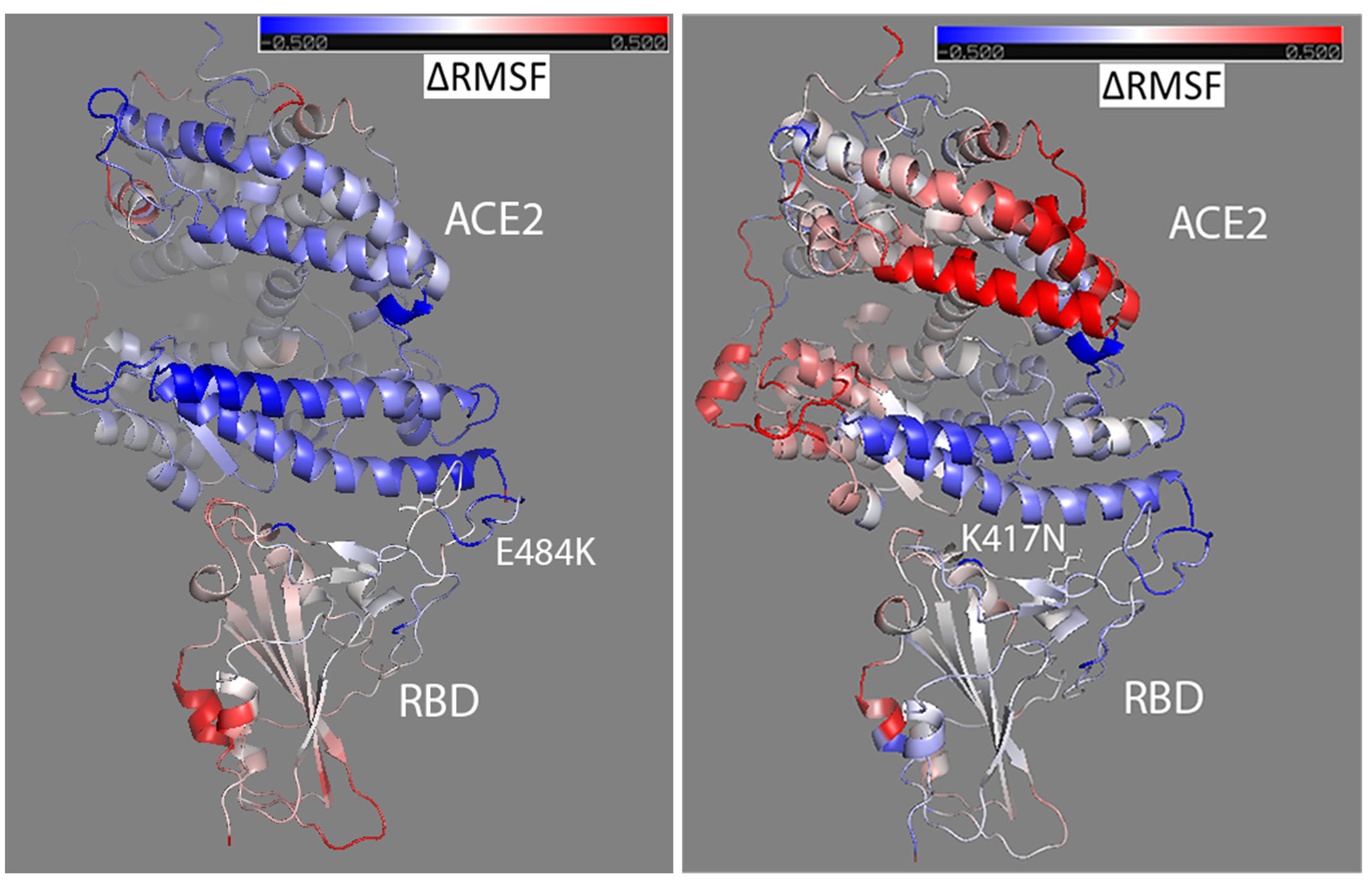

Supplement: S3 Fig — The ΔRMSF of RBD-E484K and ACE2 (Left) from RBD wildtype to variant forms mapped to the initial bound structure of ACE2&RBD-E484K; and the ΔRMSF of RBD-K417N and ACE2 (Right) mapped to its initial bound structure of the complex. Colors range from blue to white to red (with white representing no change in RMSF, blue representing RMSF decrease, and red representing RMSF increase). The ΔRMSF is in the range from -0.5 (totally blue) to 0.5 (totally red). (TIF) [file pone.0289432.s003.tif]

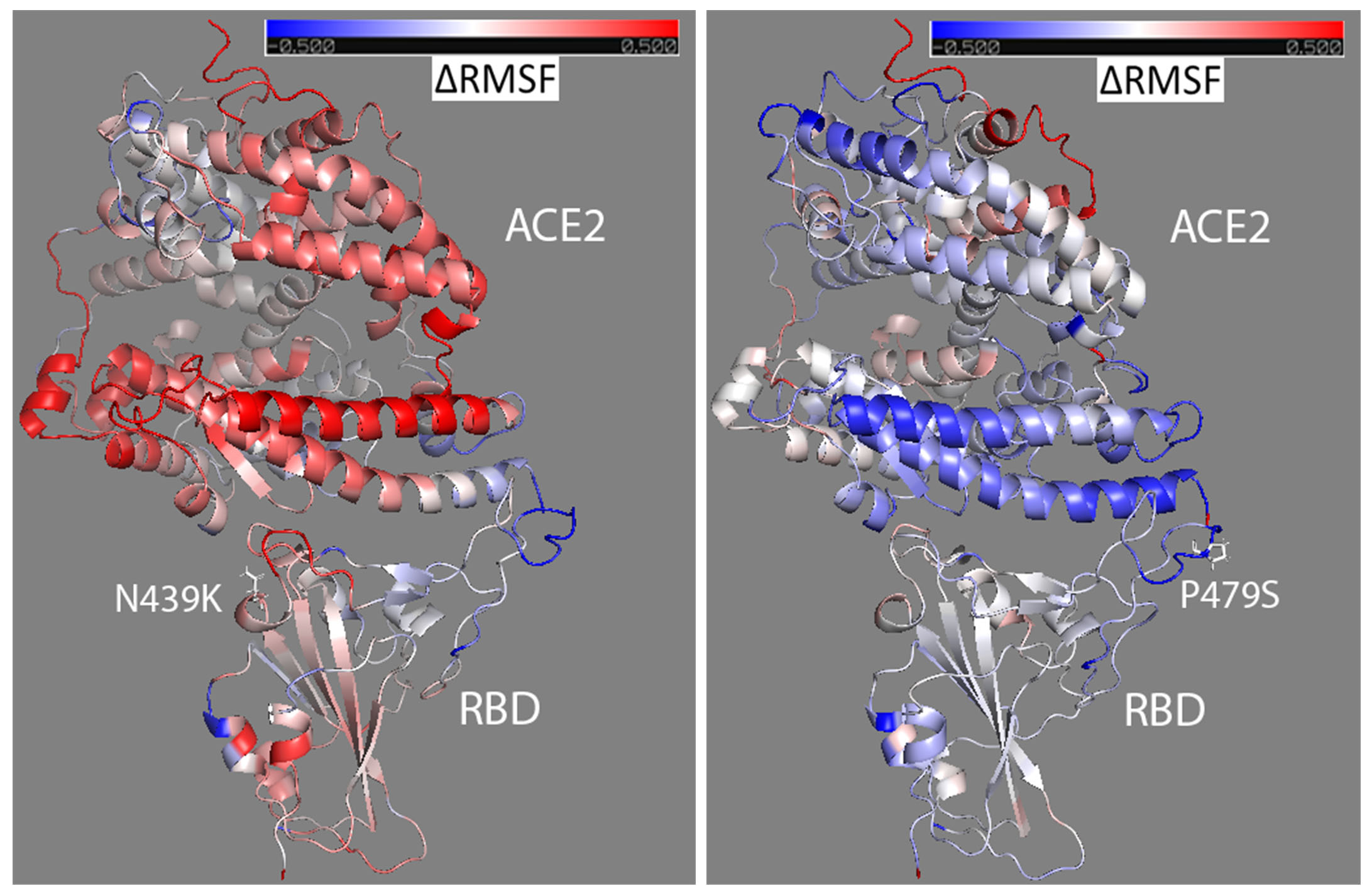

Supplement: S4 Fig — The ΔRMSF of RBD-N439K and ACE2 (Left) from RBD wildtype to variant forms mapped to the initial bound structure of ACE2&RBD-N439K; and the ΔRMSF of RBD-P479S and ACE2 (Right) mapped to its initial bound structure of the complex. Colors range from blue to white to red (with white representing no change in RMSF, blue representing RMSF decrease, and red representing RMSF increase). The ΔRMSF is in the range from -0.5 (totally blue) to 0.5 (totally red). (TIF) [file pone.0289432.s004.tif]

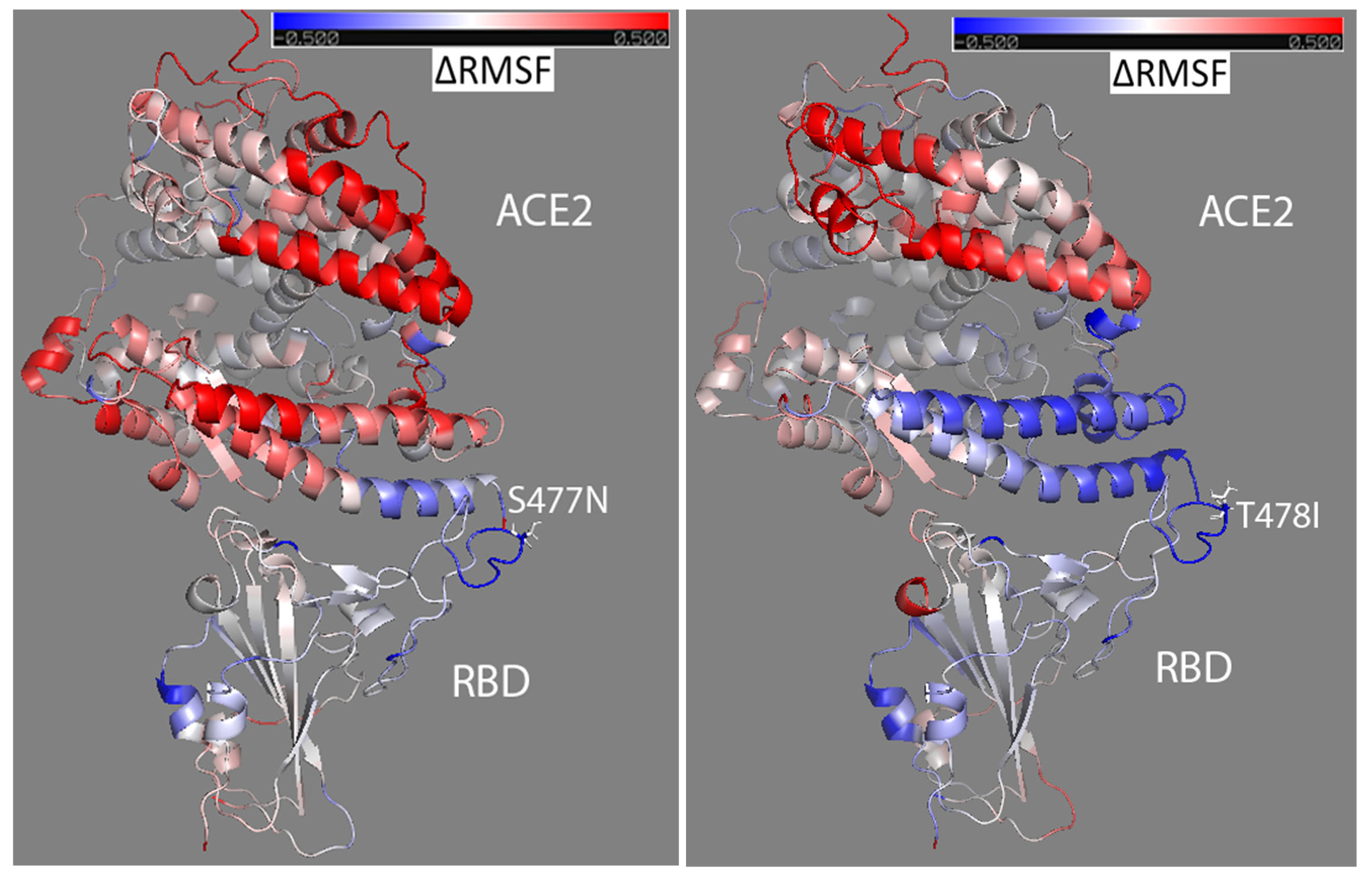

Supplement: S5 Fig — The ΔRMSF of RBD-S477N and ACE2 (Left) from RBD wildtype to variant forms mapped to the initial bound structure of ACE2&RBD-S477N; and the ΔRMSF of RBD-T478I and ACE2 (Right) mapped to its initial bound structure of the complex. Colors range from blue to white to red (with white representing no change in RMSF, blue representing RMSF decrease, and red representing RMSF increase). The ΔRMSF is in the range from -0.5 (totally blue) to 0.5 (totally red). (TIF) [file pone.0289432.s005.tif]

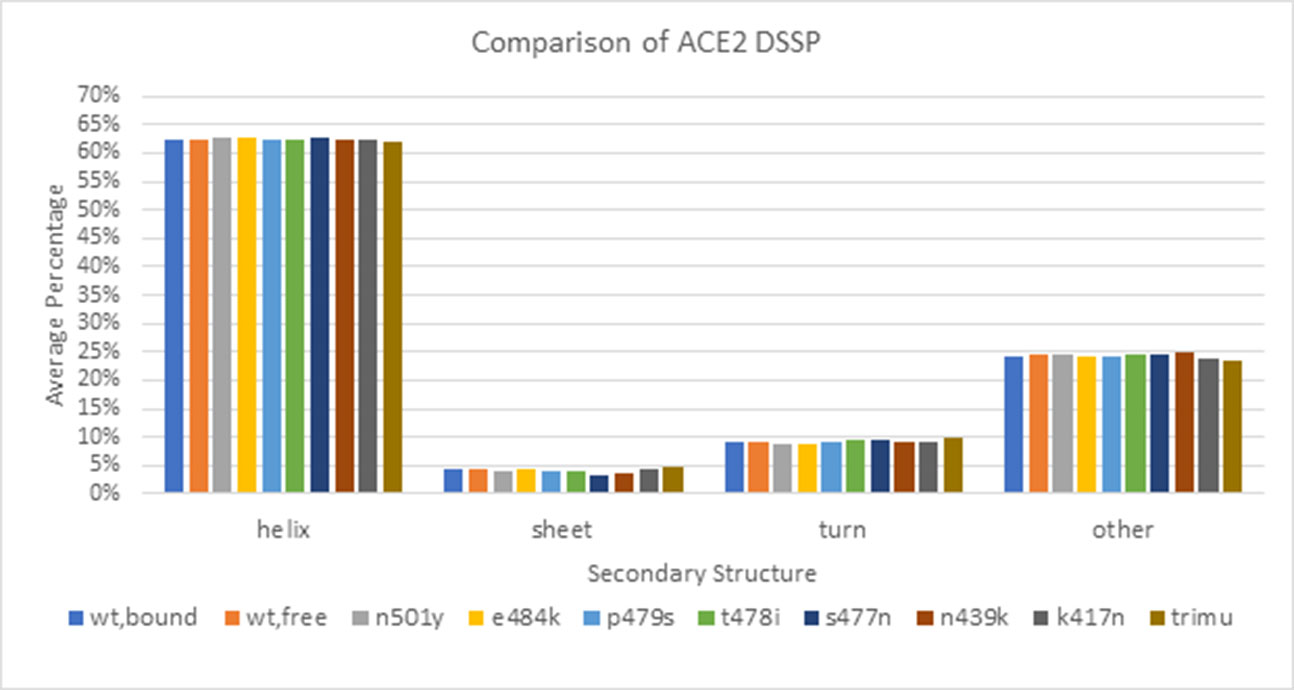

Supplement: S6 Fig — (TIF) [file pone.0289432.s006.tif]

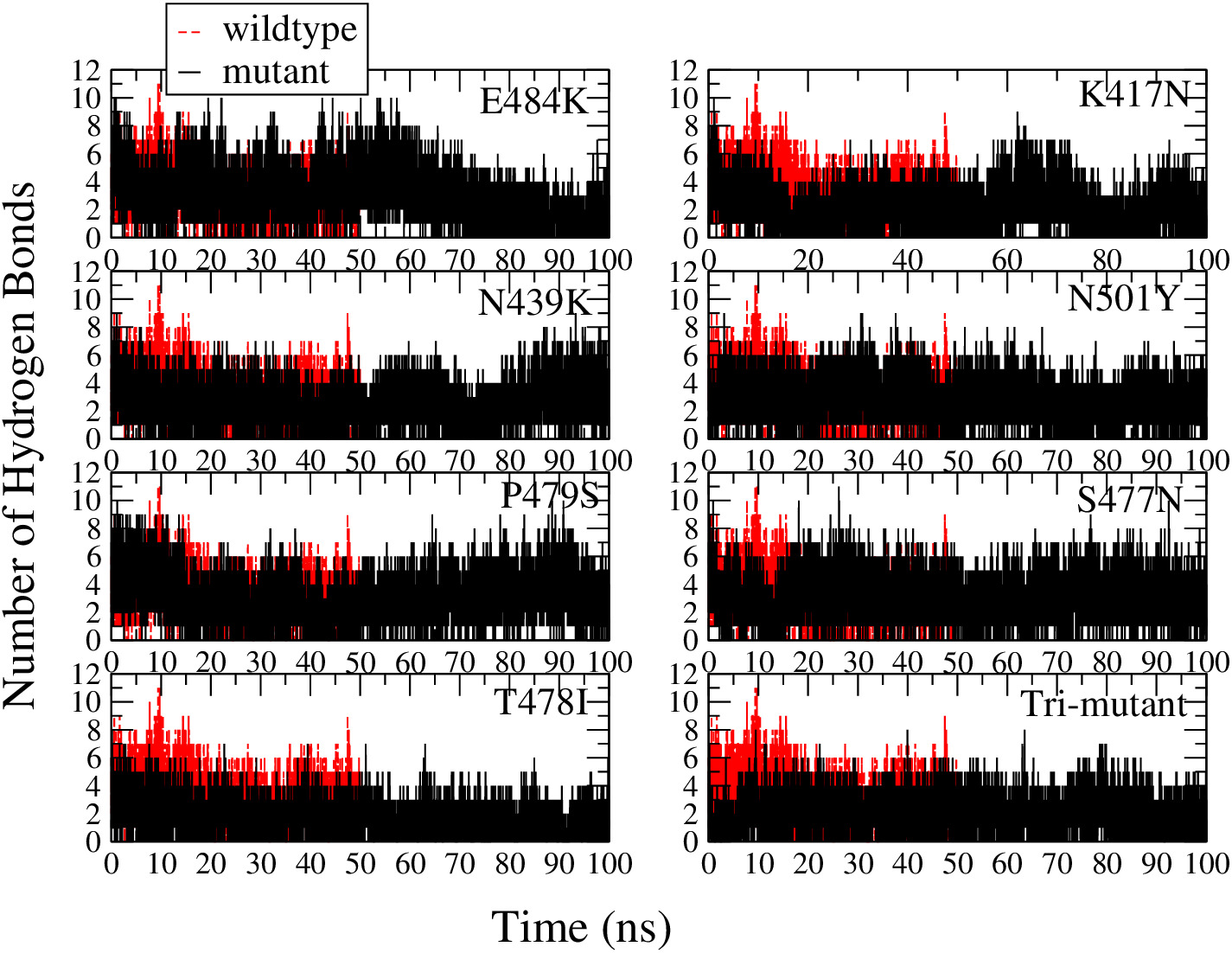

Supplement: S7 Fig — The results from mutants are shown in black in front, while red for the wildtype in back. (TIF) [file pone.0289432.s007.tif]

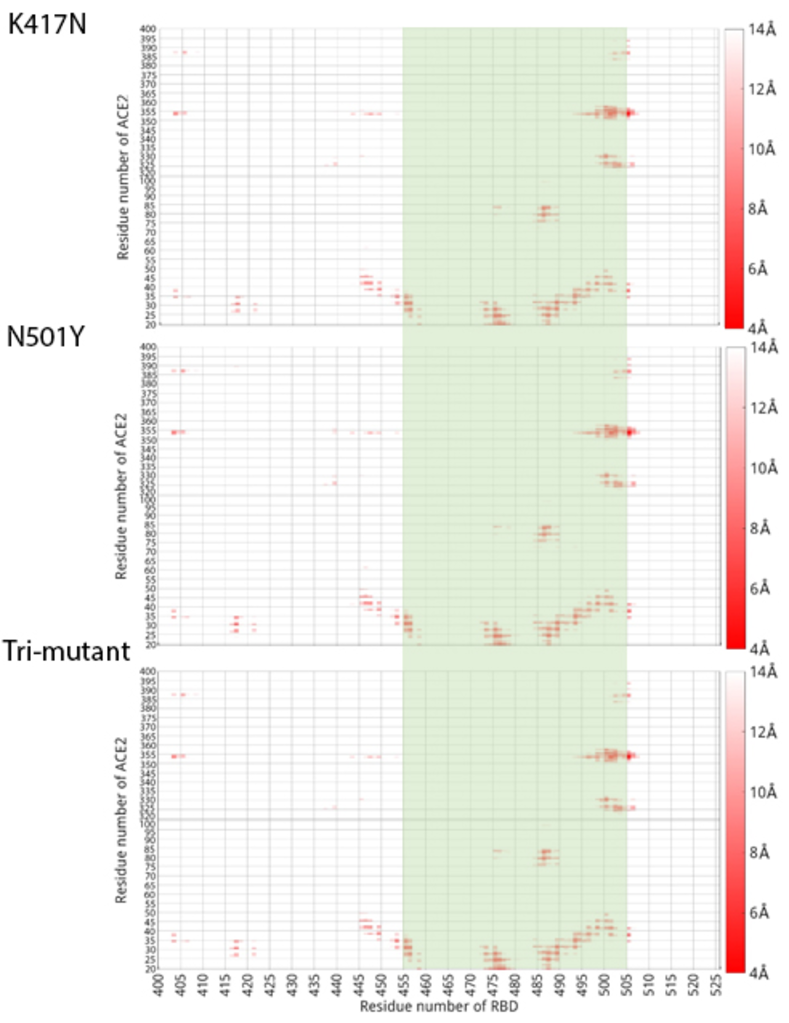

Supplement: S8 Fig — The binding region (RES455 to 505) on RBD are highlighted in light green. The color bars are shown on the right side of the distance maps, with a residue pair distance being no larger than 4 Å shown in red, while a residue pair distance larger or equal to 14 Å shown in white. (TIF) [file pone.0289432.s008.tif]

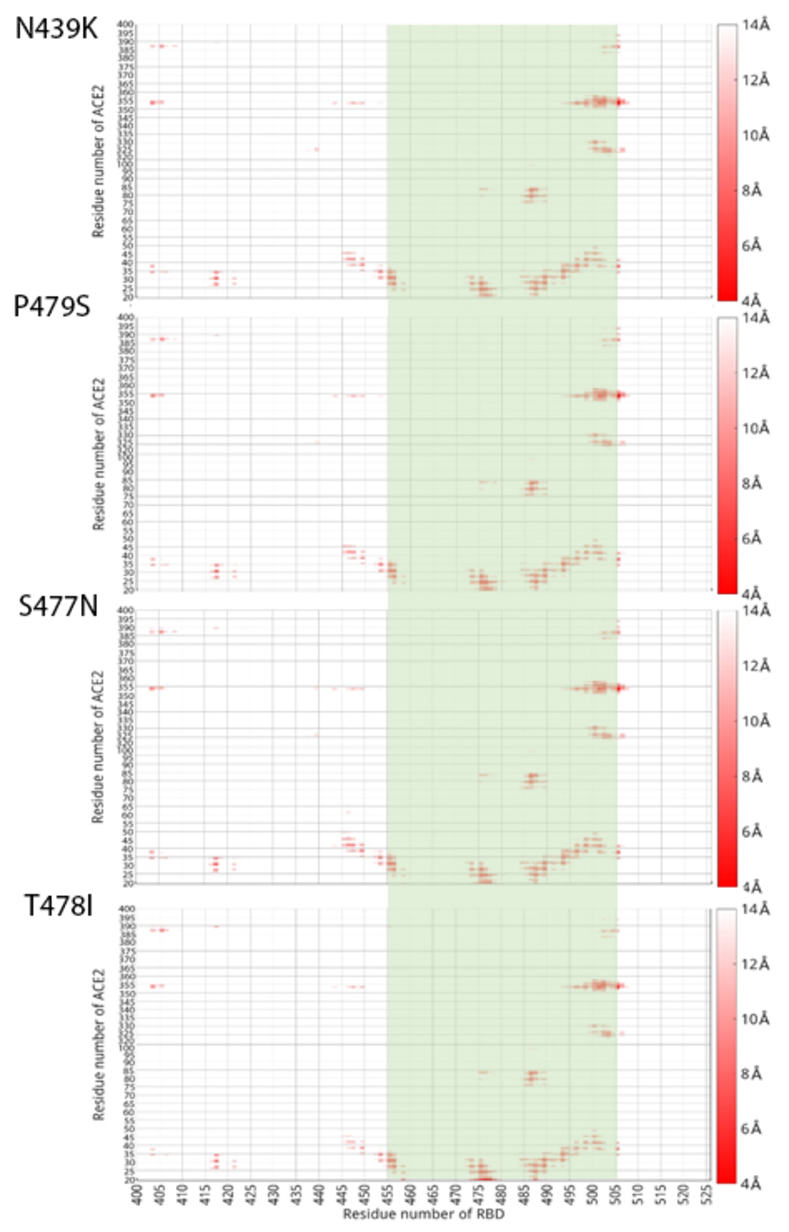

Supplement: S9 Fig — The binding region (RES455 to 505) on RBD are highlighted in light green. The color bars are shown on the right side of the distance maps, with a residue pair distance being no larger than 4 Å shown in red, while a residue pair distance larger or equal to 14 Å shown in white. (TIF) [file pone.0289432.s009.tif]

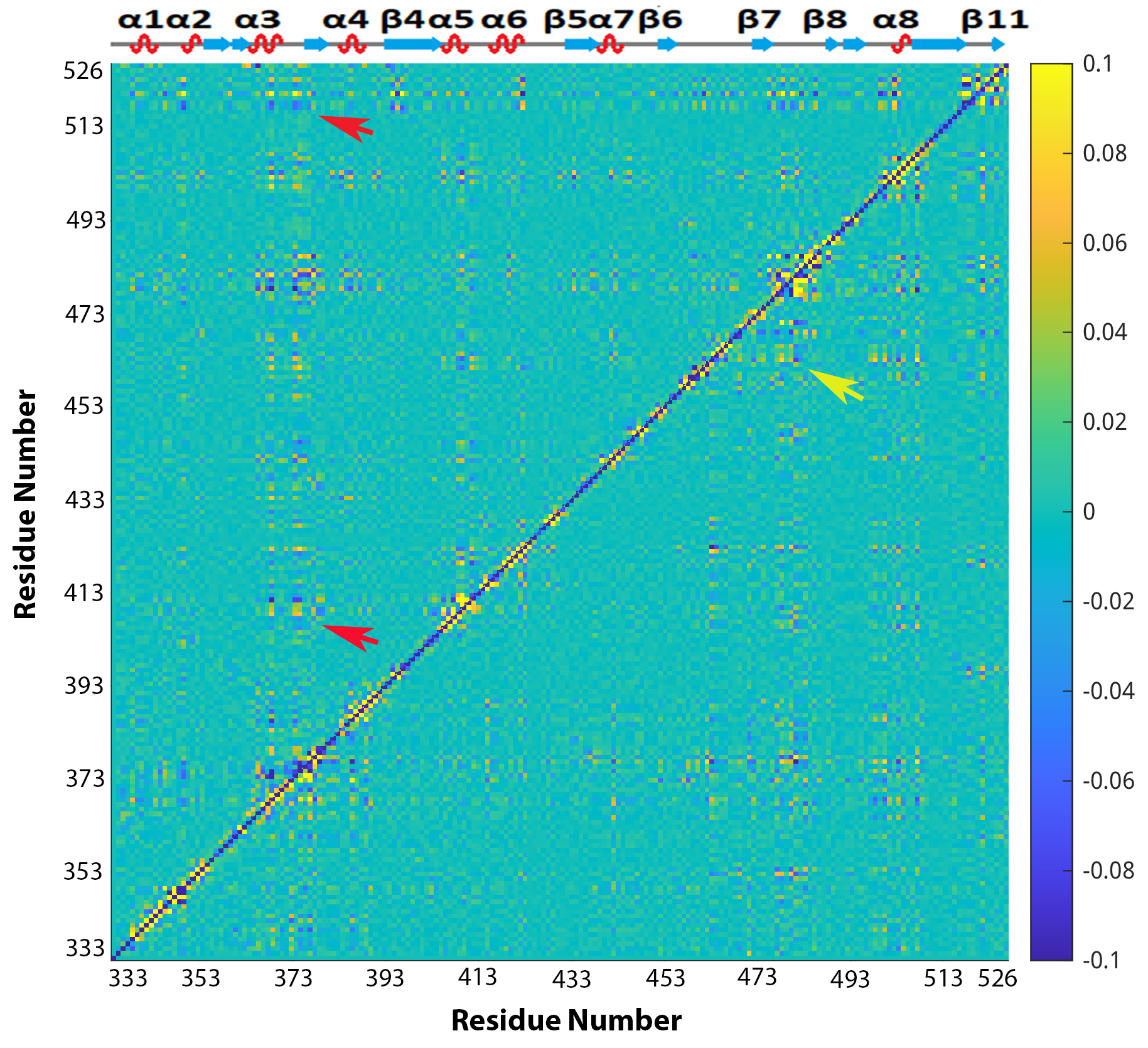

Supplement: S10 Fig — Comparison of the averaged mixed dihedral angle covariance matrix for RBD-K417N (upper-left triangle above the diagonal line) and RBD in wildtype (lower-right triangle) in bound state. The result from RBD in wildtype is shown in the lower-right triangle below the diagonal line. (TIF) [file pone.0289432.s010.tif]

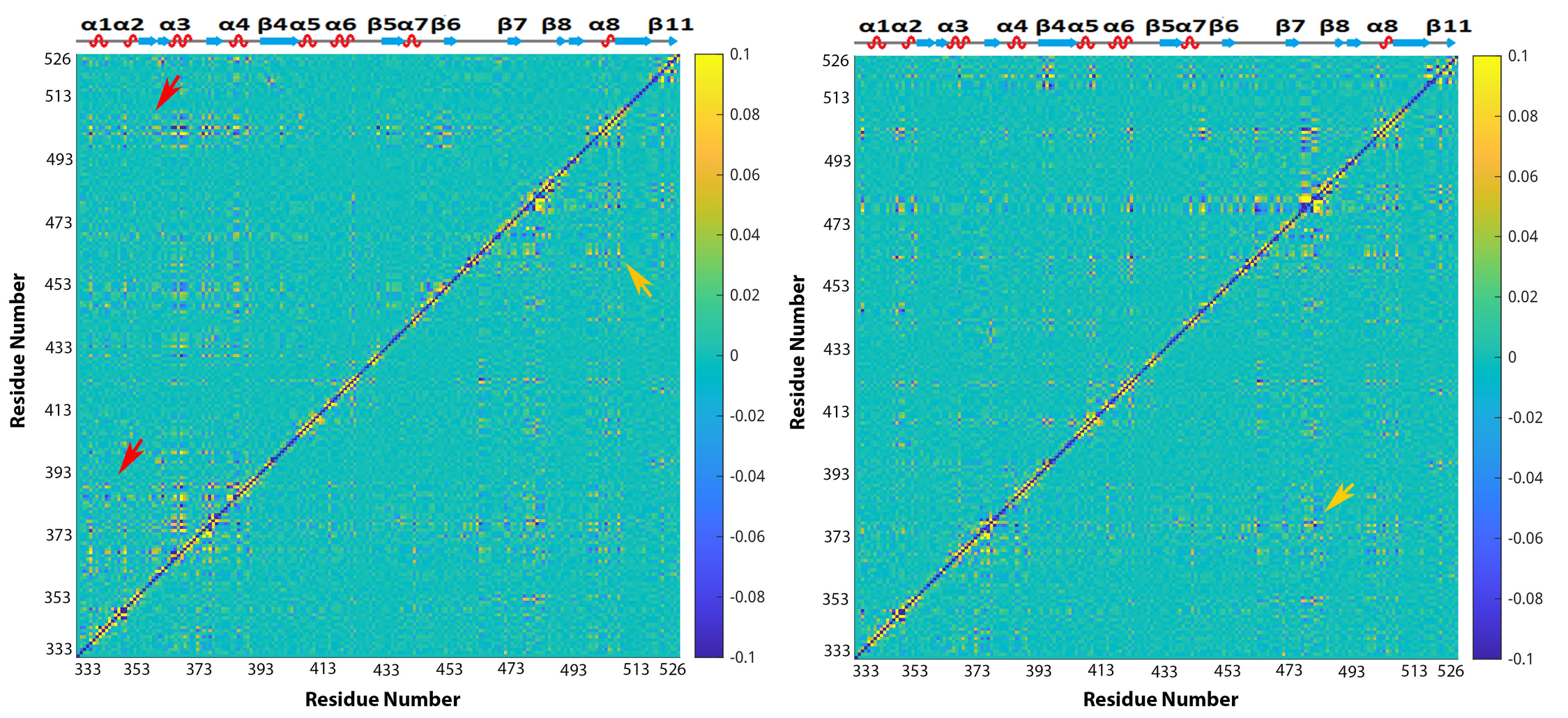

Supplement: S11 Fig — Comparison of the averaged mixed dihedral angle covariance matrix for RBD-N439K (upper-left triangle above the diagonal line) and RBD in wildtype (lower-right triangle) in bound state (Left), and RBD-P479S (upper-left triangle above the diagonal line) in comparison of RBD in wildtype (lower-right triangle) (Right). The result from RBD in wildtype is shown in the lower-right triangle below the diagonal line. (TIF) [file pone.0289432.s011.tif]

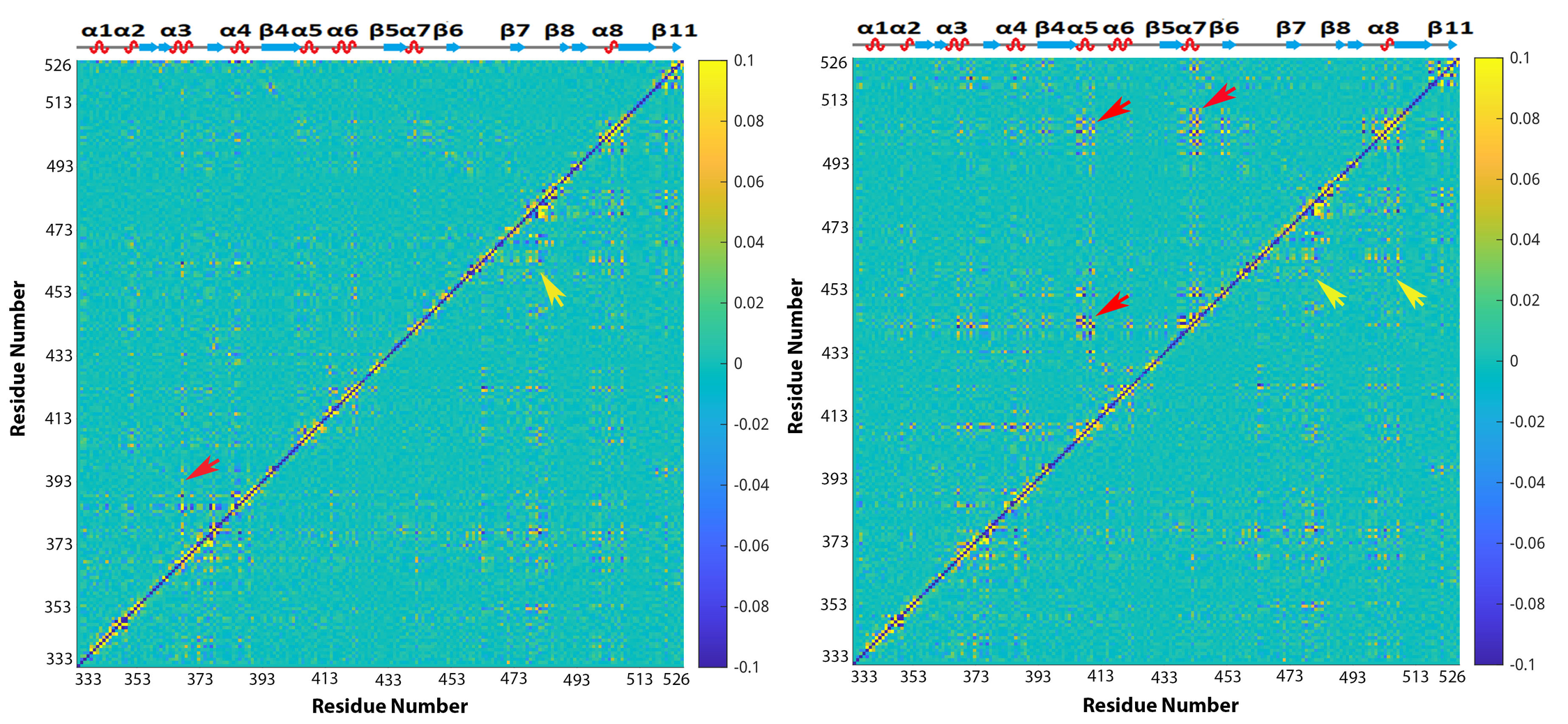

Supplement: S12 Fig — Comparison of the averaged mixed dihedral angle covariance matrix for RBD-S477N (upper-left triangle above the diagonal line) and RBD in wildtype (lower-right triangle) in bound state (Left), and RBD-T478I (upper-left triangle above the diagonal line) in comparison of RBD in wildtype (lower-right triangle) (Right). The result from RBD in wildtype is shown in the lower-right triangle below the diagonal line. (TIF) [file pone.0289432.s012.tif]

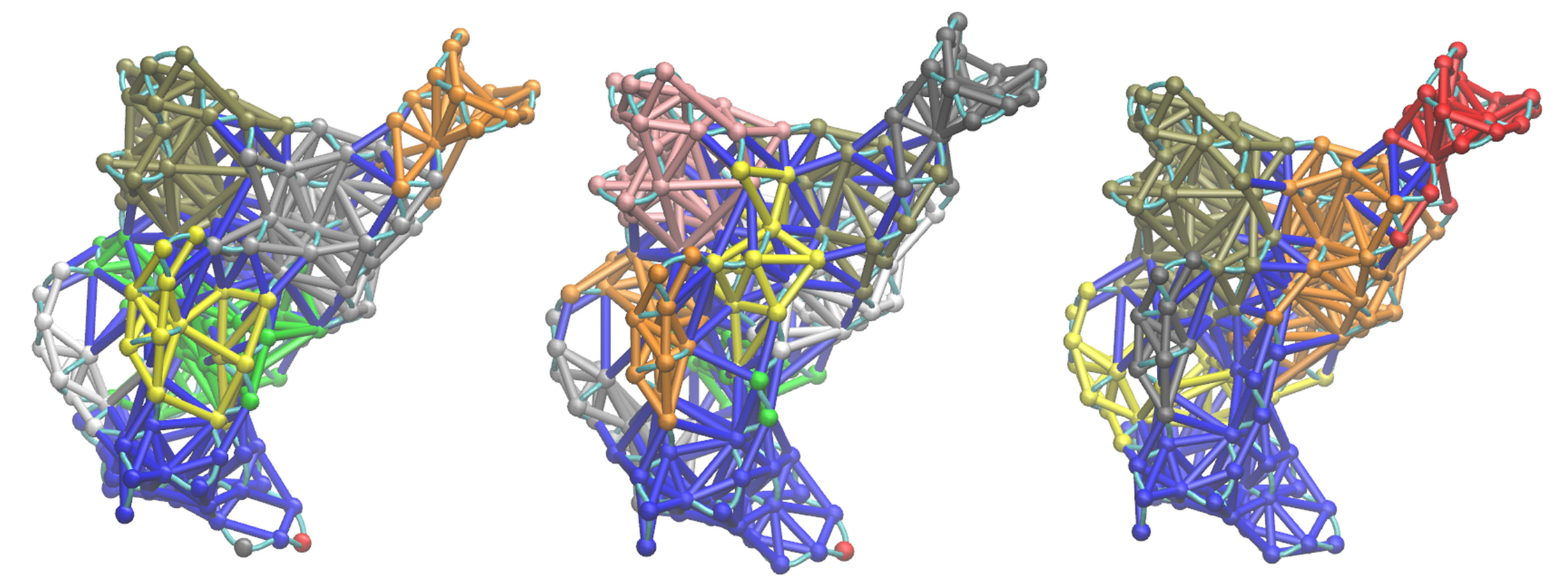

Supplement: S13 Fig — Community networks formed in the RBD-E484K(Left), in RBD-K417N (middle) and RBD-N439K (Right) based on MD simulation and dynamical network analysis. The RBD network structures are oriented in the same direction as the RBD shown in Fig 1. (TIF) [file pone.0289432.s013.tif]

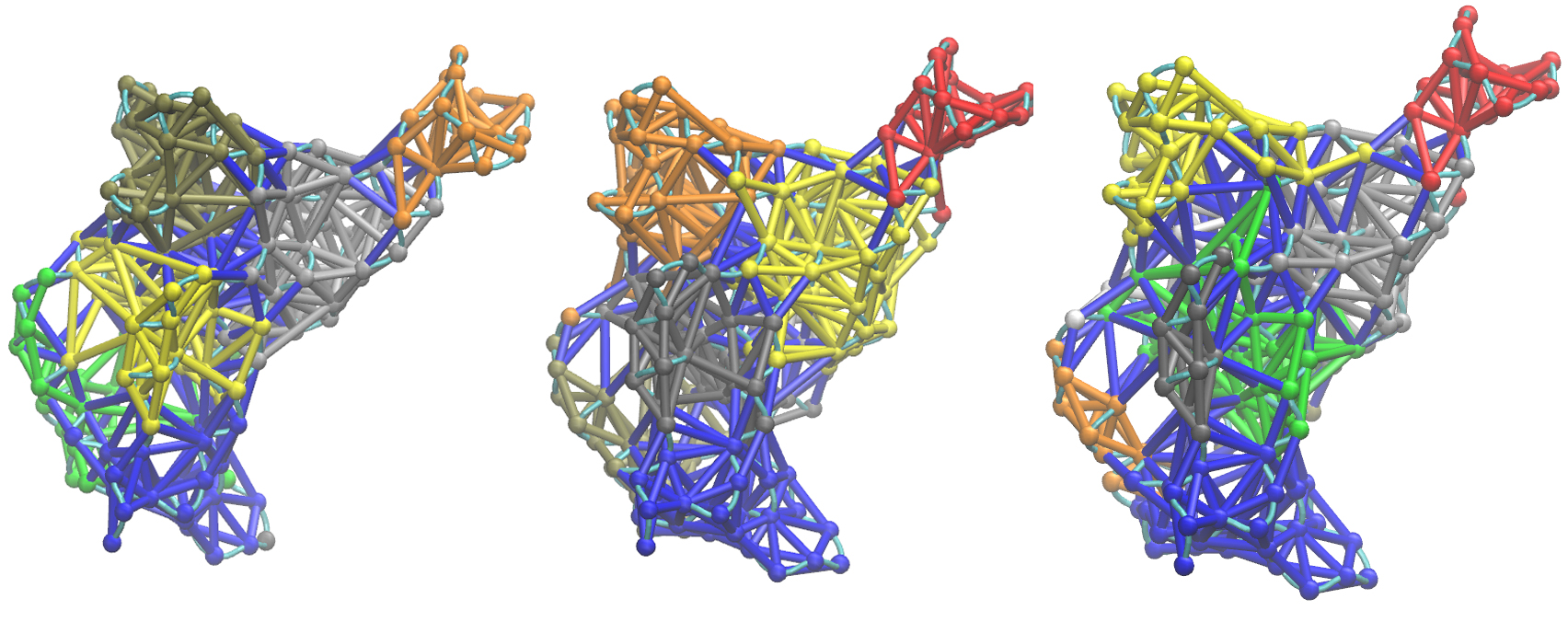

Supplement: S14 Fig — Community networks formed in the RBD-P479S(Left), in RBD-S477N (middle) and RBD-T478I (Right) based on MD simulation and dynamical network analysis. The RBD network structures are oriented in the same direction as the RBD shown in Fig 1. (TIF) [file pone.0289432.s014.tif]
