# Supplementary material for: Structure, dynamics and free energy studies on the effect of point mutations on SARS-CoV-2 spike protein binding with ACE2 receptor
Source: PLoS One. 2023 Oct 5;18(10):e0289432. doi: 10.1371/journal.pone.0289432 (PMC10553274; doi:10.1371/journal.pone.0289432)
Supplement: S1 Table — Reference [31] is listed in the main context. (DOCX) [file pone.0289432.s015.docx]

| Mutation Sites | Alpha | Beta | Gamma | Delta | Omicron |
| --- | --- | --- | --- | --- | --- |
| K417N |  | √ |  |  | √ |
| N440K |  |  |  |  | √ |
| G446S |  |  |  |  | √ |
| L452R |  |  |  | √ |  |
| S477N |  |  |  |  | √ |
| T478K |  |  |  | √ | √ |
| E484A |  |  |  |  | √ |
| E484K |  | √ | √ |  |  |
| Q493K |  |  |  |  | √ |
| G496S |  |  |  |  | √ |
| Q498R |  |  |  |  | √ |
| N501Y | √ | √ | √ |  | √ |
| Y505H |  |  |  |  | √ |
| N439K | Happened with high frequency based on genome data from Chen et al. [29] | | | | |
| P479S | Happened with high frequency based on genome data from Chen et al. [29] | | | | |
