# Supplementary material for: Structure, dynamics and free energy studies on the effect of point mutations on SARS-CoV-2 spike protein binding with ACE2 receptor
Source: PLoS One. 2023 Oct 5;18(10):e0289432. doi: 10.1371/journal.pone.0289432 (PMC10553274; doi:10.1371/journal.pone.0289432)
Supplement: S2 Table — (DOCX) [file pone.0289432.s016.docx]

| ACE2 bound with RBD mutant | Starting structure | Box size (Å^3^) | Simulation length (ns) |
| --- | --- | --- | --- |
| #1,E484K | Lan et al.[23] | 85.1x95.2x126.5 | 100 |
| #2,N439K | Lan et al.[23] | 85.1x95.2x126.6 | 100 |
| #3,N501Y | Lan et al.[23] | 85.1x95.2x126.6 | 100 |
| #4,P479S | Lan et al.[23] | 85.1x95.2x126.6 | 100 |
| #5,T478I | Lan et al.[23] | 85.1x95.2x127.2 | 100 |
| #6,K417N | Lan et al.[23] | 85.1x95.2x126.5 | 100 |
| #7,S477N | Lan et al.[23] | 85.1x95.2x127.1 | 100 |
| #8,N501Y-E484K-K417N | Lan et al.[23] | 85.1x95.2x126.9 | 100 |
| #9, RBD wildtype | Lan et al.[23] | 65.7x73.3x74.3 | 100 |
| #10, ACE2 | Lan et al.[23] | 93.3x94.8x78.8 | 100 |
| FEP simulations, |  | | |
| Mutant site | Starting Structure | Box size (Å^3^) | Simulation length (ns) |
| #1,E484K | Lan et al.[23] | 82.4x92.2x134.6 | 3 |
|  |  | 62.3x70.6x77.8 | 3 |
| #2,N439K | Lan et al.[23] | 82.4x92.2x134.9 | 3 |
|  |  | 64.2x70.6x78.1 | 3 |
| #3,N501Y | Lan et al.[23] | 82.5x92.3x134.7 | 3 |
|  |  | 64.4x70.7x78.1 | 3 |
| #4,K417N | Lan et al.[23] | 82.4x92.2x134.8 | 3 |
|  |  | 64.3x70.6x77.8 | 3 |
| #5,S477N | Lan et al.[23] | 82.4x92.1x134.9 | 3 |
|  |  | 64.3x70.6x77.9 | 3 |
| #6,P479S | Lan et al.[23] | 82.5x92.3x134.6 | 3 |
|  |  | 64.2x70.6x77.8 | 3 |
| #7,T478I | Lan et al.[23] | 82.5x92.3x134.6 | 3 |
|  |  | 64.3x70.6x77.9 | 3 |
