# Supplementary material for: Structure, dynamics and free energy studies on the effect of point mutations on SARS-CoV-2 spike protein binding with ACE2 receptor
Source: PLoS One. 2023 Oct 5;18(10):e0289432. doi: 10.1371/journal.pone.0289432 (PMC10553274; doi:10.1371/journal.pone.0289432)
Supplement: S3 Table — Only hydrogen bonds with an occupancy higher than 10% are shown. The residue pairs which form hydrogen bonds on ACE2 and RBD wildtype are highlighted in blue in the ACE2 and RBD mutant jobs. (DOCX) [file pone.0289432.s017.docx]

| Bound with ACE2 jobs | RES#(RBD)-RES#(ACE2) | Occupancy  (%) | Bound with ACE2 jobs | RBD-ACE2 | Occupancy  (%) |
| --- | --- | --- | --- | --- | --- |
| RBD-K417N | THR500-ASP355 | 42.36 | RBD-N439K | LYS417-ASP30 | 45.3 |
|  | GLY502-LYS353 | 38.55 |  | GLY502-LYS353 | 43.4 |
|  | ASN487-TYR83 | 36.28 |  | ASN487-TYR83 | 38.2 |
|  | TYR505-GLU37 | 24.61 |  | THR500-ASP355 | 32.8 |
|  | GLN493-GLU35 | 23.32 |  | TYR505-GLU37 | 30.9 |
|  | TYR449-ASP38 | 15.63 |  | GLN493-GLU35 | 27.6 |
| Bound with ACE2 jobs | RBD-ACE2 | Occupancy  (%) | Bound with ACE2 jobs | RBD-ACE2 | Occupancy  (%) |
| RBD-T478I | LYS417-ASP30 | 42.9 | RBD-N501Y | GLY502-LYS353 | 54.3 |
|  | GLY502-LYS353 | 40.3 |  | LYS417-ASP30 | 44.4 |
|  | ASN487-TYR83 | 35.8 |  | ASN487-TYR83 | 41.7 |
|  | GLN493-GLU35 | 10.2 |  | TYR505-GLU37 | 31.7 |
|  |  |  |  | THR500-ASP355 | 30.0 |
|  |  |  |  | GLN493-GLU35 | 22.0 |
|  |  |  |  | THR500-TYR41 | 13.7 |
|  |  |  |  | GLN498-GLN42 | 10.2 |
| Bound with ACE2 jobs | RBD-ACE2 | Occupancy  (%) | Bound with ACE2 jobs | RBD-ACE2 | Occupancy  (%) |
| RBD-P479S | LYS417-ASP30 | 52.4 | RBD-S477N | LYS417-ASP30 | 46.2 |
|  | GLY502-LYS353 | 46.2 |  | GLY502-LYS353 | 44.9 |
|  | TYR505-GLU37 | 39.2 |  | ASN487-TYR83 | 40.0 |
|  | ASN487-TYR83 | 37.8 |  | TYR505-GLU37 | 33.1 |
|  | GLN493-GLU35 | 26.1 |  | GLN493-GLU35 | 25.7 |
|  | THR500-TYR41 | 17.3 |  | THR500-ASP355 | 23.6 |
|  | TYR449-ASP38 | 14.6 |  | THR500-TYR41 | 17.0 |
|  | GLN498-LYS353 | 10.9 |  | GLN493-LYS31 | 12.5 |
|  |  |  |  | GLN498-GLN42 | 11.9 |
| Bound with ACE2 jobs | RES#(RBD)-RES# (ACE2) | Occupancy  (%) | Bound with ACE2 jobs | RBD-ACE2 | Occupancy  (%) |
| RBD-E484K | LYS417-ASP30 | 40.6 | RBD -N501Y-E484K-K417N | GLY502-LYS353 | 52.0 |
|  | ASN487-TYR83 | 40.3 |  | ASN487-TYR83 | 33.8 |
|  | TYR505-GLU37 | 31.9 |  | THR500-ASP355 | 25.6 |
|  | GLY502-LYS353 | 30.8 |  | GLN493-GLU35 | 23.2 |
|  | THR500-ASP355 | 30.7 |  | TYR505-GLU37 | 19.1 |
|  | GLN498-GLN42 | 21.5 |  | THR500-TYR41 | 11.8 |
|  | GLN493-GLU35 | 17.6 |  |  |  |
|  | GLN498-LYS353 | 17.4 |  |  |  |
|  | TYR449-ASP38 | 17.4 |  |  |  |
|  | THR500-TYR41 | 13.4 |  |  |  |
|  | GLN498-GLN42 | 12.3 |  |  |  |
| Bound with ACE2 jobs | RES#(RBD)-RES# (ACE2) | Occupancy  (%) |  |  |  |
| RBD-wt | GLY502-LYS353 | 42.05 |  |  |  |
|  | ASN487-TYR83 | 41.46 |  |  |  |
|  | LYS417-ASP30 | 34.23 |  |  |  |
|  | GLN498-LYS353 | 27.21 |  |  |  |
|  | TYR449-ASP38 | 24.67 |  |  |  |
|  | TYR505-GLU37 | 22.53 |  |  |  |
|  | THR500-TYR41 | 22.35 |  |  |  |
|  | GLN493-GLU35 | 21.14 |  |  |  |
|  | GLN498-GLN42 | 19.43 |  |  |  |
