# Supplementary material for: Structure, dynamics and free energy studies on the effect of point mutations on SARS-CoV-2 spike protein binding with ACE2 receptor
Source: PLoS One. 2023 Oct 5;18(10):e0289432. doi: 10.1371/journal.pone.0289432 (PMC10553274; doi:10.1371/journal.pone.0289432)
Supplement: S1 File — (PDF) [file pone.0289432.s018.pdf]

Supplemental Material to

Structure, dynamics and free energy studies on the effect of point mutations on SARS-CoV-2 spike protein binding with ACE2 receptor

George Rucker[1], Hong Qin[2], Liqun Zhang[3*]

1. Chemical Engineering Department, Tennessee Technological University, Cookeville, TN, 38501
2. Computer Science department, University of Tennessee Chattanooga, Chattanooga, TN 37403
3. Chemical Engineering Department, University of Rhode Island, Kingston, RI, 02881

Figures

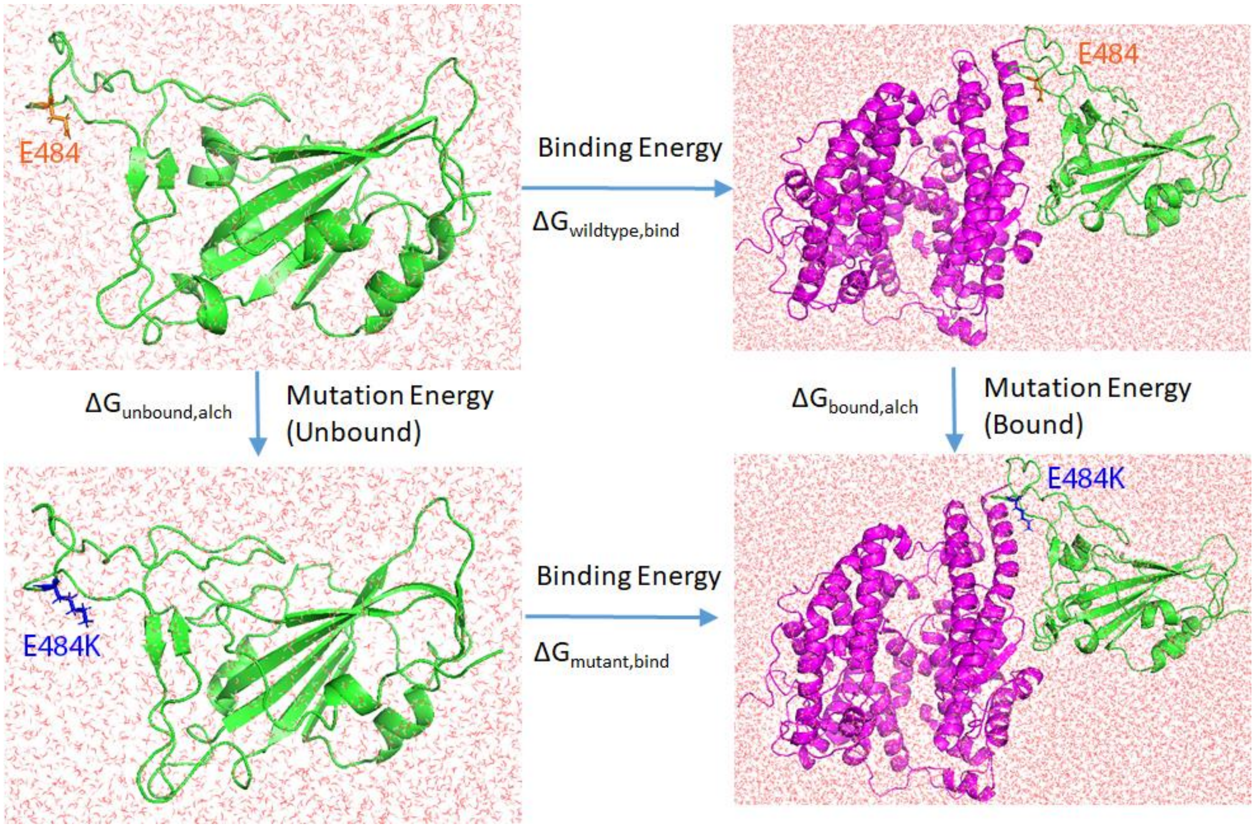

Fig S1.

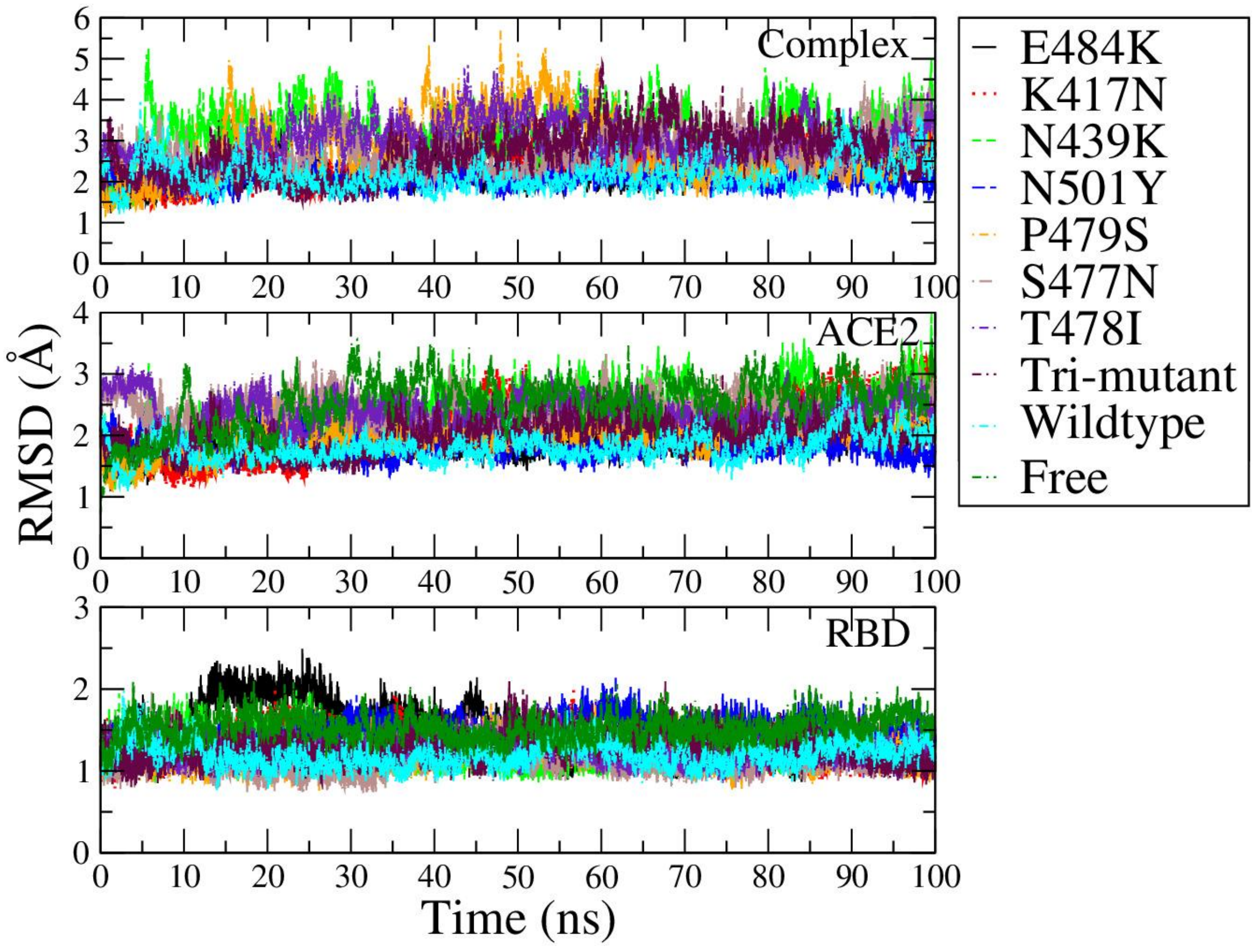
Complex
ACE2
RBD
RMSD (Å)
Time (ns)
E484K
K417N
N439K
N501Y
P479S
S477N
T478I
Tri-mutant
Wildtype
Free

Fig S2.

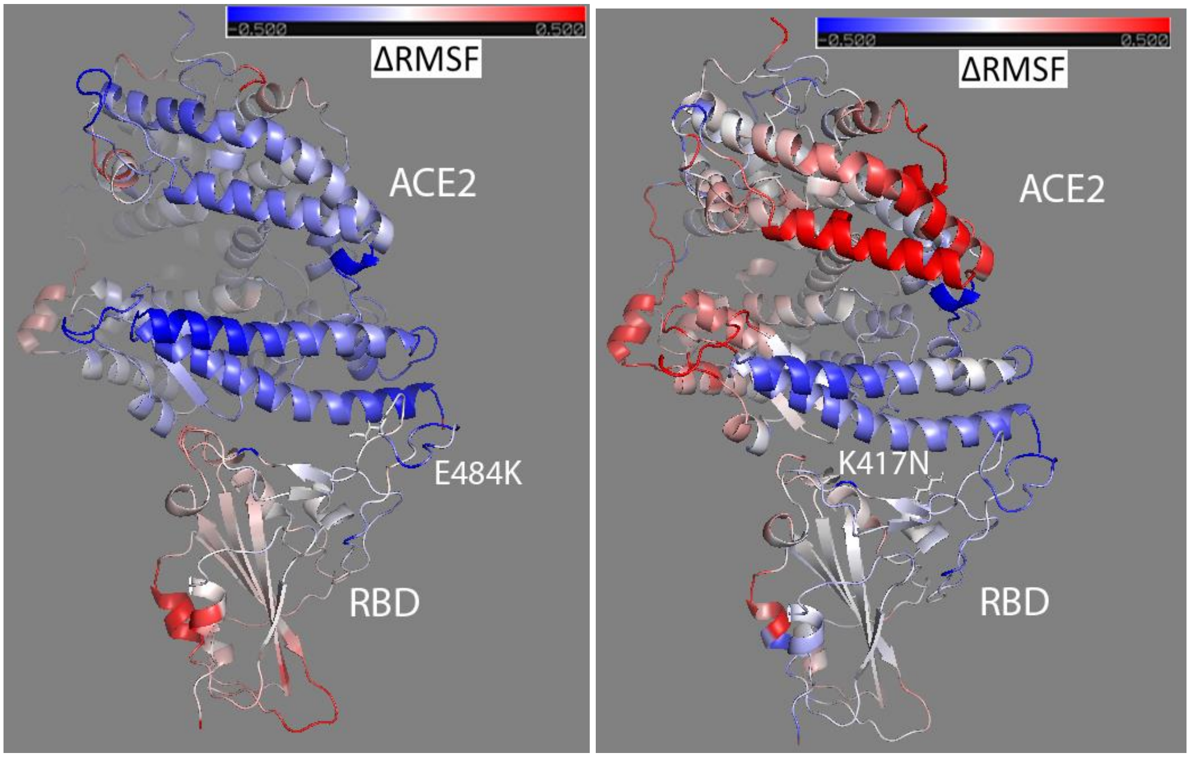
-0.500
0.500
ΔRMSF
ACE2
E484K
RBD
-0.500
0.500
ΔRMSF
ACE2
K417N
RBD

Fig S3.

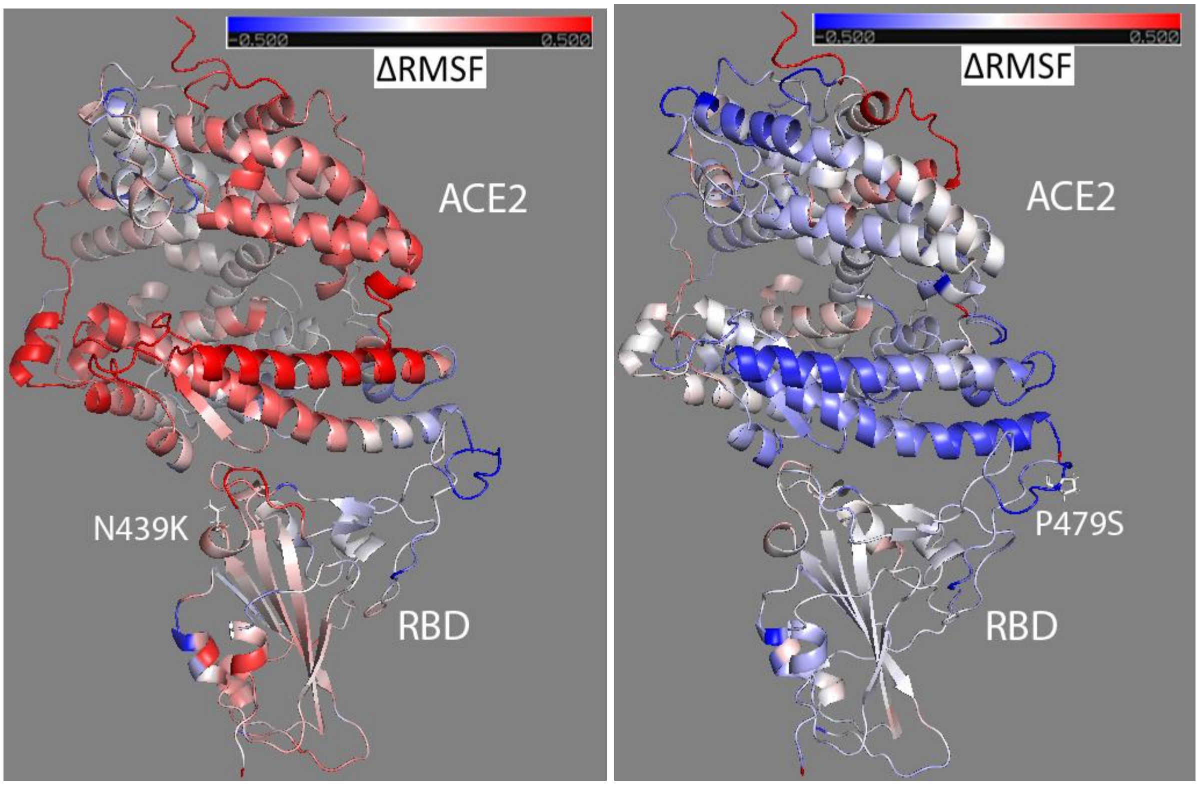
-0.500
0.500
ΔRMSF
ACE2
N439K
RBD
-0.500
0.500
ΔRMSF
ACE2
P479S
RBD

Fig S4.

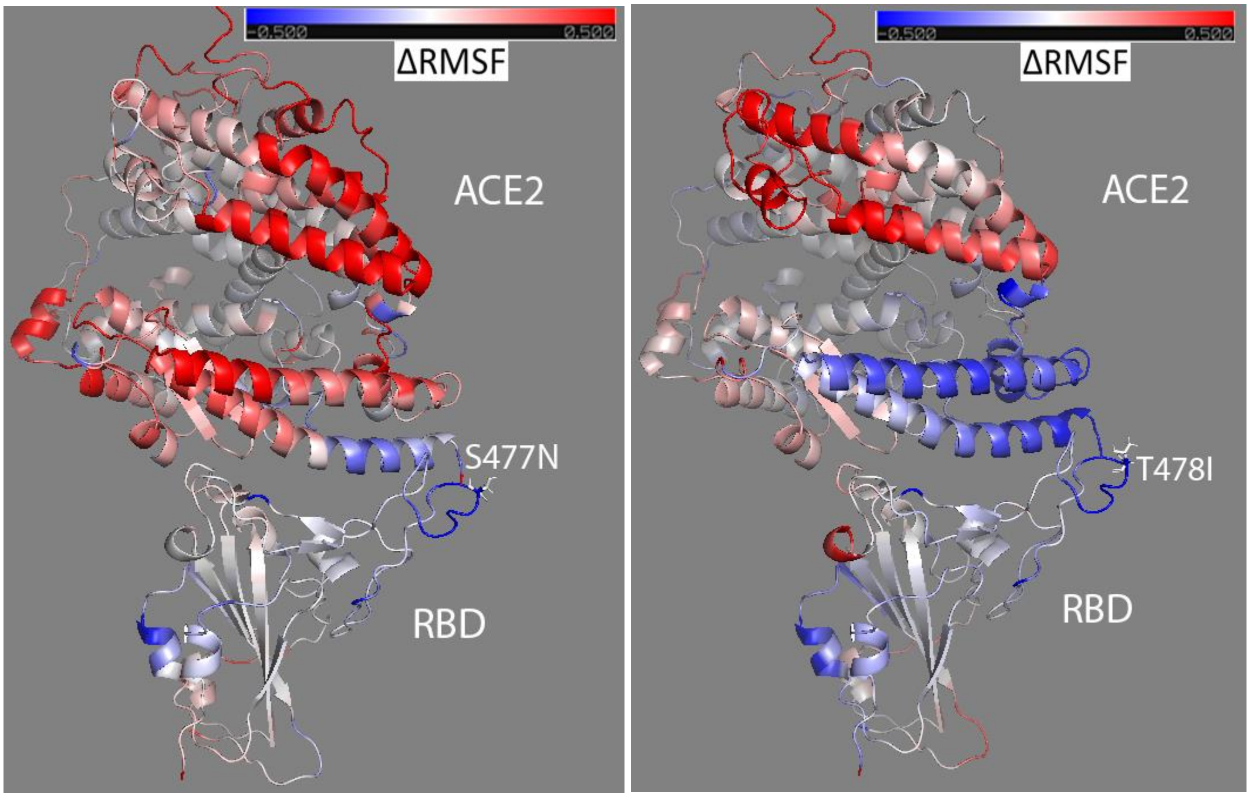

-0.500
0.500
ΔRMSF
ACE2
S477N
RBD
-0.500
0.500
ΔRMSF
ACE2
T478I
RBD

Fig S5.

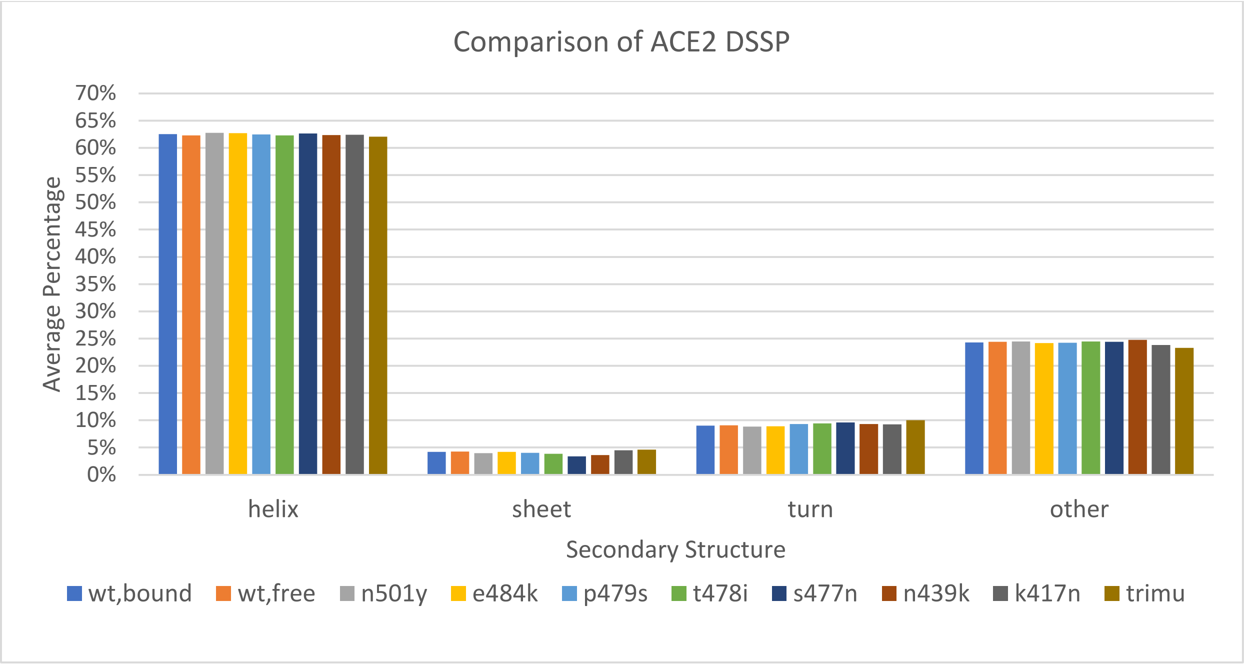

Comparison of ACE2 DSSP
70%
65%
60%
55%
50%
45%
40%
35%
30%
25%
20%
15%
10%
5%
0%
Average Percentage
helix
sheet
turn
other
Secondary Structure
wt,bound
wt,free
n501y
e484k
p479s
t478i
s477n
n439k
k417n
trimu

Fig S6.

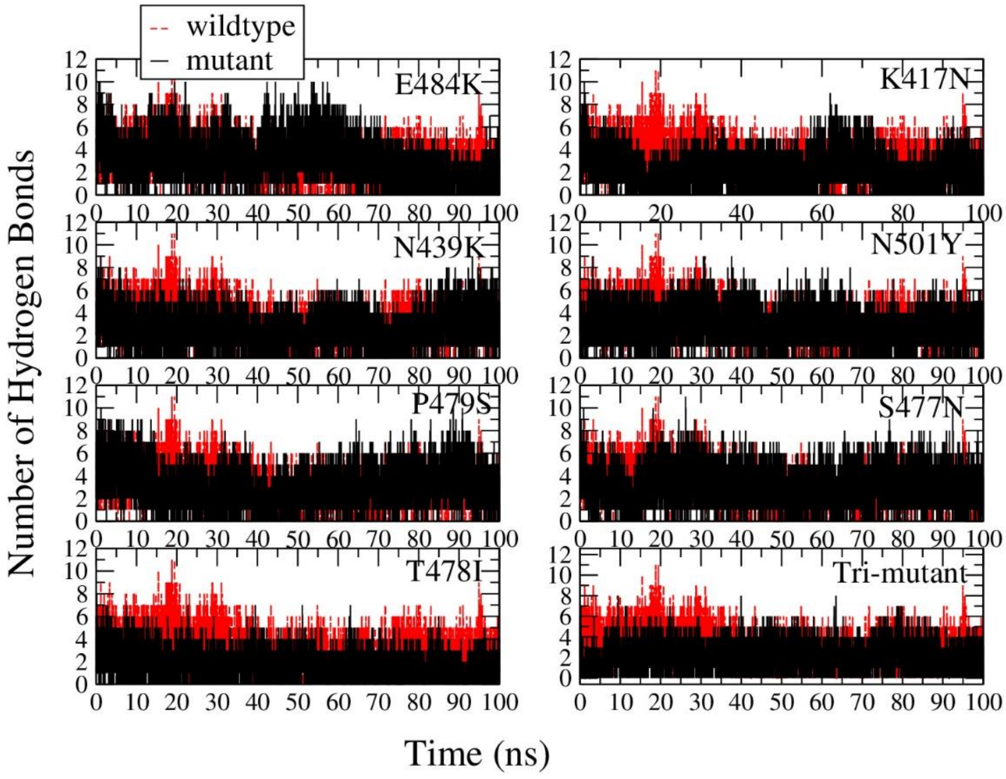

wildtype
mutant
E484K
K417N
N439K
N501Y
P479S
S477N
T478I
Tri-mutant
Number of Hydrogen Bonds
Time (ns)

Fig S7.

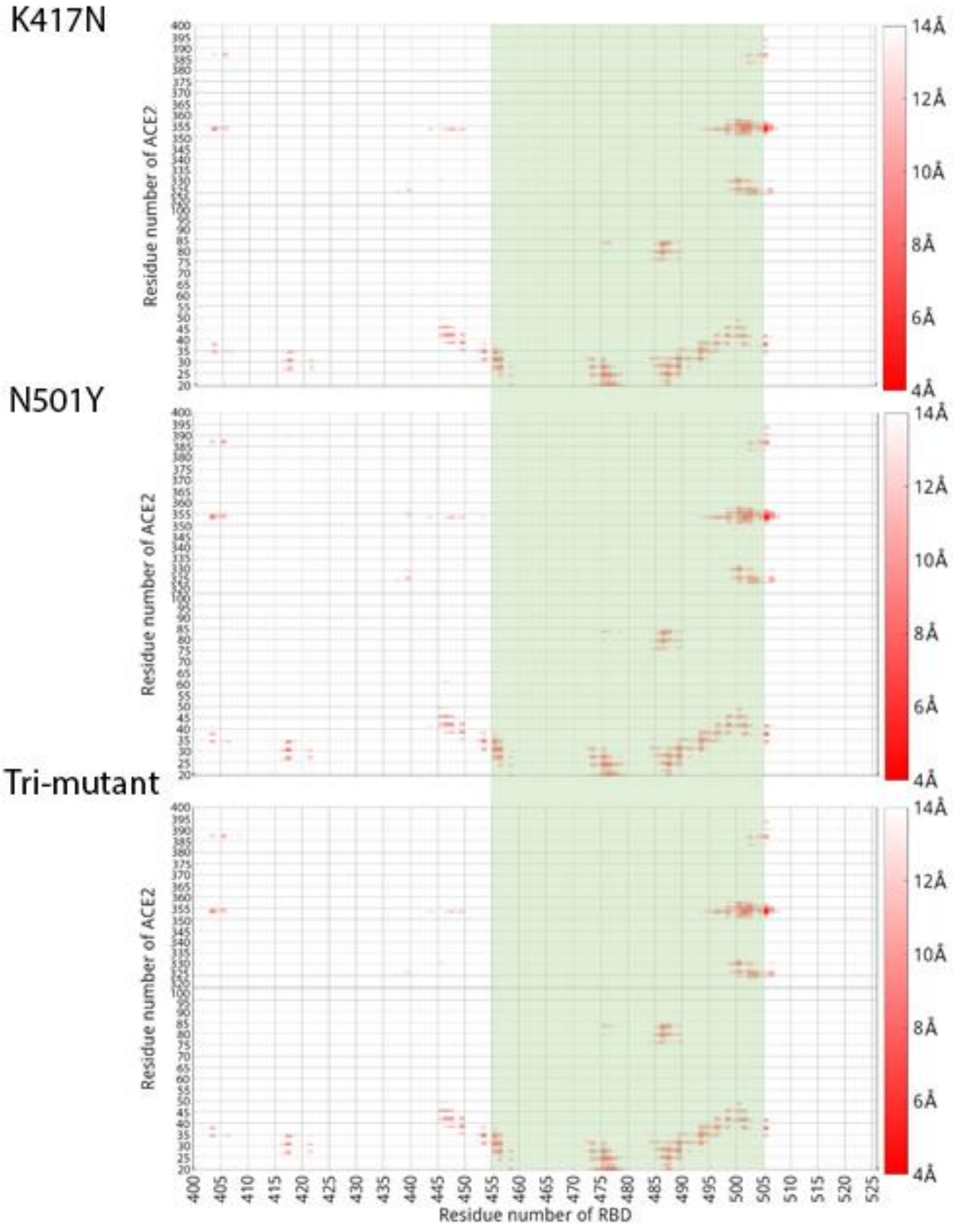

K417N
N501Y
Tri-mutant
Residue number of ACE2
Residue number of RBD
14Å
12Å
10Å
8Å
6Å
4Å

Fig S8.

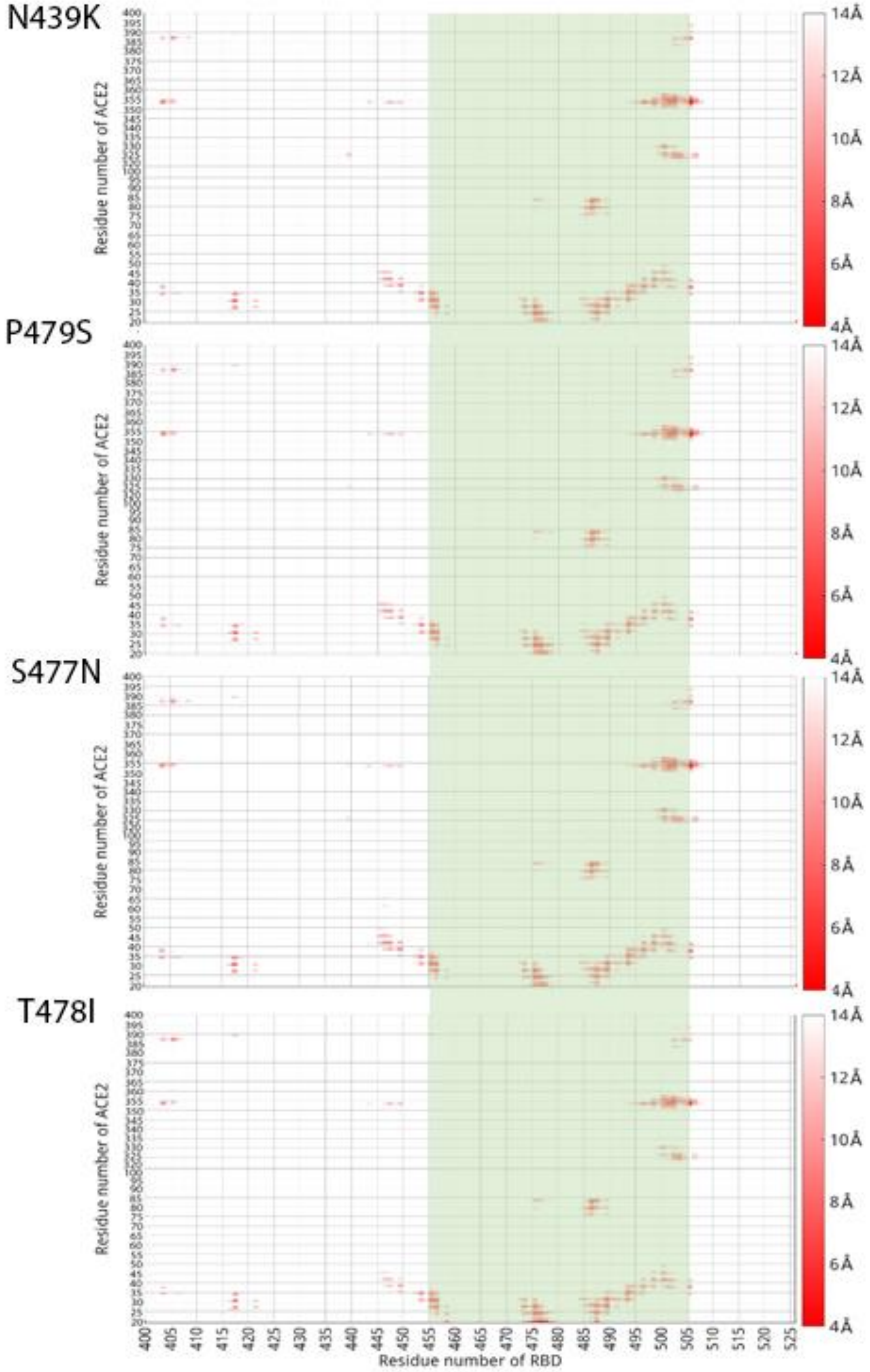

N439K
P479S
S477N
T478I
Residue number of ACE2
Residue number of RBD
14Å
12Å
10Å
8Å
6Å
4Å

Fig S9.

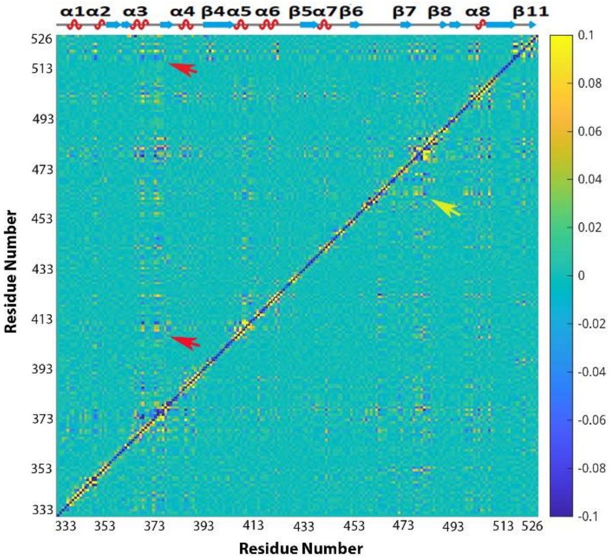

Fig S10.

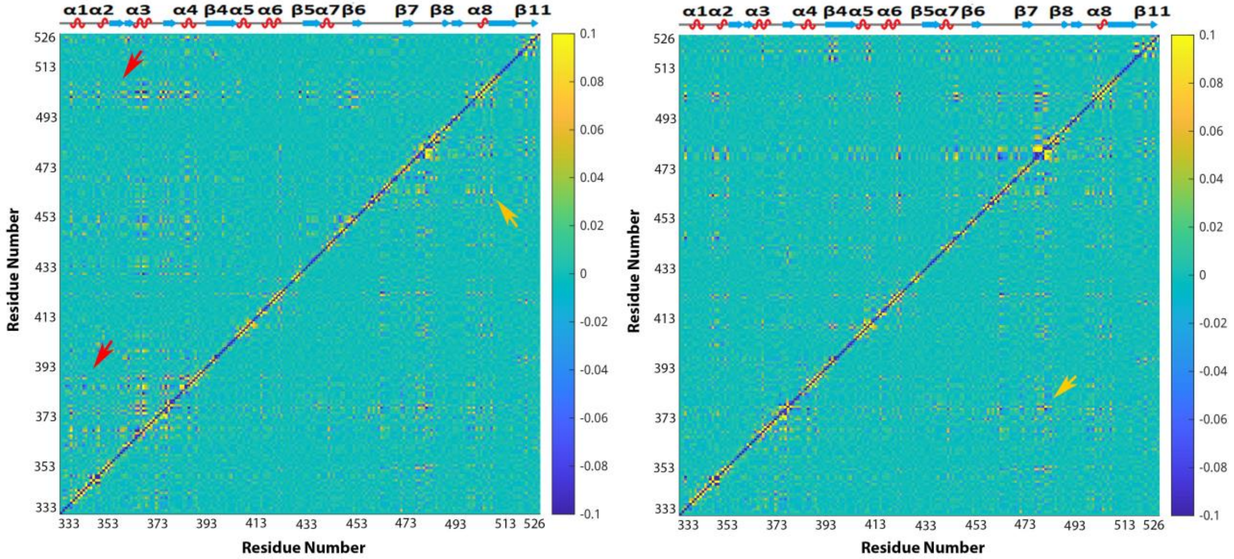

Fig S11.

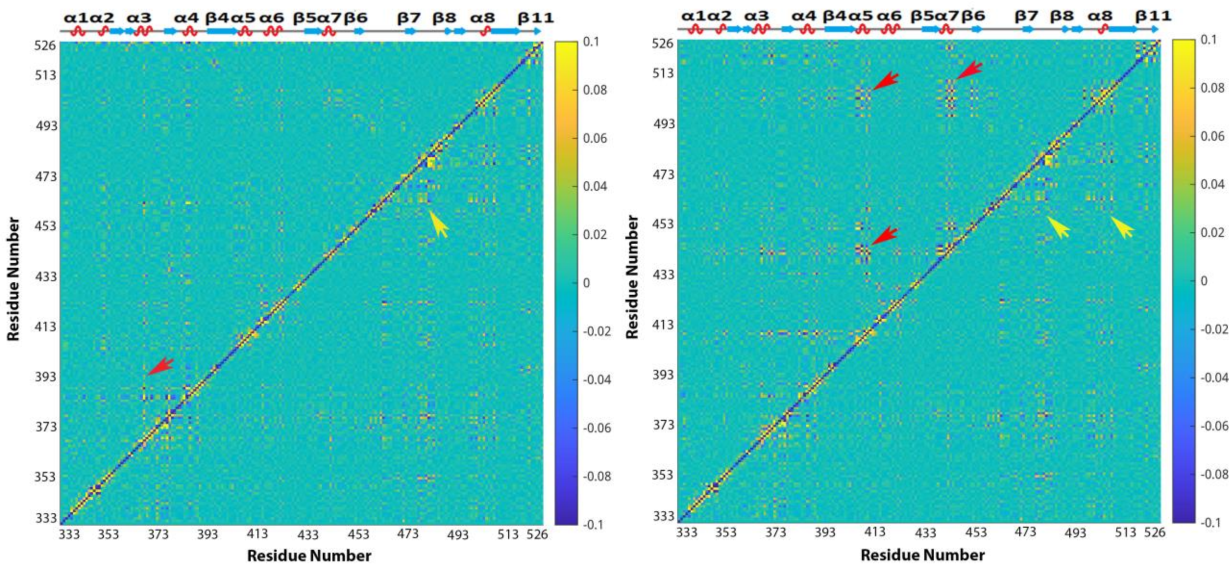

Fig S12.

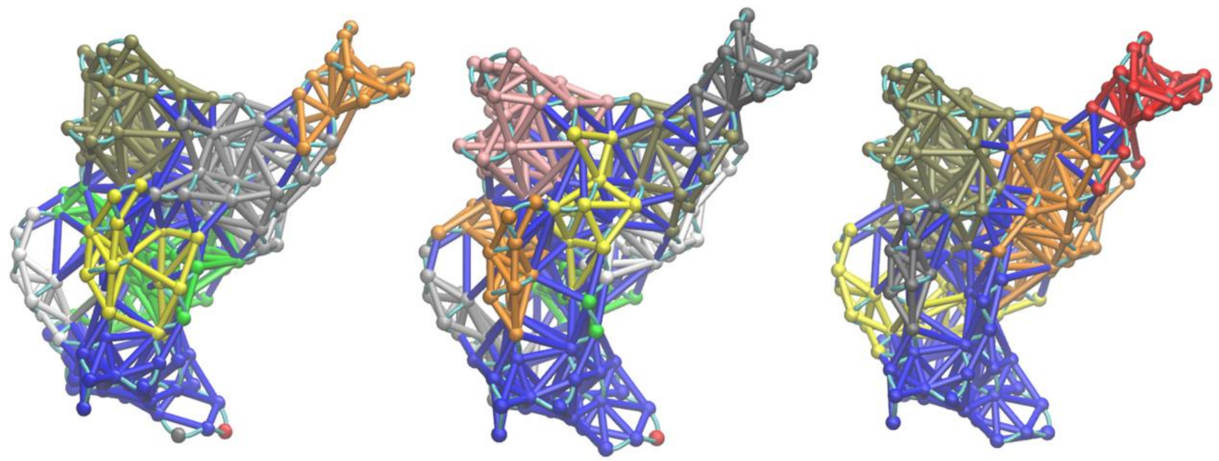

Fig S13.

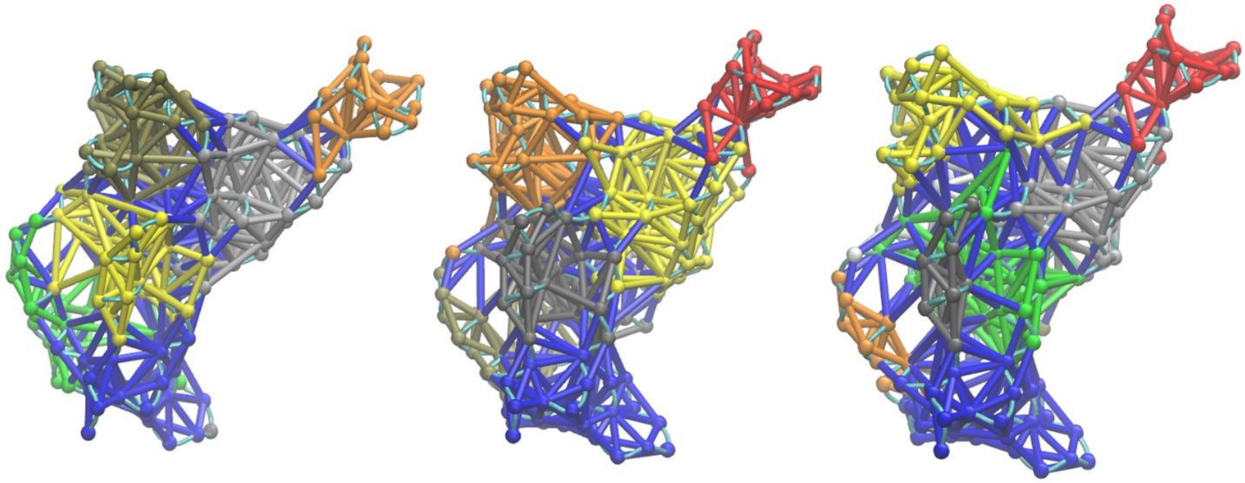

Fig S14.

Tables

Table S1.

Mutation Sites	Alpha	Beta	Gamma	Delta	Omicron
K417N		√			√
N440K					√
G446S					√
L452R				√	
S477N					√
T478K				√	√
E484A					√
E484K		√	√		
Q493K					√
G496S					√
Q498R					√
N501Y	√	√	√		√
Y505H					√
N439K	Happened with high frequency based on genome data from Chen et al. [29]				
P479S	Happened with high frequency based on genome data from Chen et al. [29]				

Table S2.

ACE2 bound with RBD mutant	Starting structure	Box size (Å^3)	Simulation length (ns)
#1,E484K	Lan et al.[23]	85.1x95.2x126.5	100
#2,N439K	Lan et al.[23]	85.1x95.2x126.6	100
#3,N501Y	Lan et al.[23]	85.1x95.2x126.6	100
#4,P479S	Lan et al.[23]	85.1x95.2x126.6	100
#5,T478I	Lan et al.[23]	85.1x95.2x127.2	100
#6,K417N	Lan et al.[23]	85.1x95.2x126.5	100
#7,S477N	Lan et al.[23]	85.1x95.2x127.1	100
#8,N501Y-E484K-K417N	Lan et al.[23]	85.1x95.2x126.9	100
#9, RBD wildtype	Lan et al.[23]	65.7x73.3x74.3	100
#10, ACE2	Lan et al.[23]	93.3x94.8x78.8	100
FEP simulations,			
Mutant site	Starting Structure	Box size (Å^3)	Simulation length (ns)
#1,E484K	Lan et al.[23]	82.4x92.2x134.6	3
		62.3x70.6x77.8	3
#2,N439K	Lan et al.[23]	82.4x92.2x134.9	3
		64.2x70.6x78.1	3
#3,N501Y	Lan et al.[23]	82.5x92.3x134.7	3
		64.4x70.7x78.1	3
#4,K417N	Lan et al.[23]	82.4x92.2x134.8	3
		64.3x70.6x77.8	3
#5,S477N	Lan et al.[23]	82.4x92.1x134.9	3
		64.3x70.6x77.9	3
#6,P479S	Lan et al.[23]	82.5x92.3x134.6	3
		64.2x70.6x77.8	3
#7,T478I	Lan et al.[23]	82.5x92.3x134.6	3
		64.3x70.6x77.9	3

Table S3.

Bound with ACE2 jobs	RES#(RBD)-RES#(ACE2)	Occupancy (%)	Bound with ACE2 jobs	RBD-ACE2	Occupancy (%)
RBD-K417N	THR500-ASP355	42.36	RBD-N439K	LYS417-ASP30	45.3
	GLY502-LYS353	38.55		GLY502-LYS353	43.4
	ASN487-TYR83	36.28		ASN487-TYR83	38.2
	TYR505-GLU37	24.61		THR500-ASP355	32.8
	GLN493-GLU35	23.32		TYR505-GLU37	30.9
	TYR449-ASP38	15.63		GLN493-GLU35	27.6
Bound with ACE2 jobs	RBD-ACE2	Occupancy (%)	Bound with ACE2 jobs	RBD-ACE2	Occupancy (%)
RBD-T478I	LYS417-ASP30	42.9	RBD-N501Y	GLY502-LYS353	54.3

	GLY502-LYS353	40.3		LYS417-ASP30	44.4
	ASN487-TYR83	35.8		ASN487-TYR83	41.7
	GLN493-GLU35	10.2		TYR505-GLU37	31.7
				THR500-ASP355	30.0
				GLN493-GLU35	22.0
				THR500-TYR41	13.7
				GLN498-GLN42	10.2
Bound with ACE2 jobs	RBD-ACE2	Occupancy (%)	Bound with ACE2 jobs	RBD-ACE2	Occupancy (%)
RBD-P479S	LYS417-ASP30	52.4	RBD-S477N	LYS417-ASP30	46.2
	GLY502-LYS353	46.2		GLY502-LYS353	44.9
	TYR505-GLU37	39.2		ASN487-TYR83	40.0
	ASN487-TYR83	37.8		TYR505-GLU37	33.1
	GLN493-GLU35	26.1		GLN493-GLU35	25.7
	THR500-TYR41	17.3		THR500-ASP355	23.6
	TYR449-ASP38	14.6		THR500-TYR41	17.0
	GLN498-LYS353	10.9		GLN493-LYS31	12.5
				GLN498-GLN42	11.9
Bound with ACE2 jobs	RES#(RBD)-RES# (ACE2)	Occupancy (%)	Bound with ACE2 jobs	RBD-ACE2	Occupancy (%)
RBD-E484K	LYS417-ASP30	40.6	RBD -N501Y-E484K-K417N	GLY502-LYS353	52.0
	ASN487-TYR83	40.3		ASN487-TYR83	33.8
	TYR505-GLU37	31.9		THR500-ASP355	25.6
	GLY502-LYS353	30.8		GLN493-GLU35	23.2
	THR500-ASP355	30.7		TYR505-GLU37	19.1
	GLN498-GLN42	21.5		THR500-TYR41	11.8
	GLN493-GLU35	17.6			
	GLN498-LYS353	17.4			
	TYR449-ASP38	17.4			
	THR500-TYR41	13.4			
	GLN498-GLN42	12.3			
Bound with ACE2 jobs	RES#(RBD)-RES# (ACE2)	Occupancy (%)			
RBD-wt	GLY502-LYS353	42.05			
	ASN487-TYR83	41.46			
	LYS417-ASP30	34.23			
	GLN498-LYS353	27.21			
	TYR449-ASP38	24.67			
	TYR505-GLU37	22.53			
	THR500-TYR41	22.35			
	GLN493-GLU35	21.14			
	GLN498-GLN42	19.43			
